# The limitations of fully threaded screws in isolated percutaneous transarticular screw fixation of C1/C2

**DOI:** 10.1038/s41598-022-10447-x

**Published:** 2022-04-20

**Authors:** Leon-Gordian Koepke, Annika Heuer, Martin Stangenberg, Marc Dreimann, Jörg Beyerlein, Christian Schaefer, Lennart Viezens

**Affiliations:** 1 Division of Spine Surgery, Department of Trauma Surgery and Orthopaedic Surgery, University Medical Center Hamburg-Eppendorf, Martinistrasse 52, 20246 Hamburg, Germany; 2 ATOS Klinik Fleetinsel, Hamburg, Germany; 3 Department of Spine Surgery, Klinikum Bad Bramstedt, Bad Bramstedt, Germany; 4 Division of Orthopaedics, Department of Trauma Surgery and Orthopaedic Surgery, University Medical Center Hamburg-Eppendorf, Hamburg, Germany

**Keywords:** Outcomes research, Trauma, Fracture repair, Geriatrics, Quality of life

## Abstract

Demographic aging accompanied by increased falls inevitably leads to an increased incidence of atlantoaxial instabilities (AAI). Minimally invasive surgical procedures decrease the perioperative risk and regarding the treatment of AAI, percutaneous transarticular screw fixation of C1/C2 was more frequently considered in the past. This study aims to investigate the outcome of patients treated for AAI by isolated percutaneous transarticular screw fixation of C1/C2 (IPTSFC1/C2) using 3.5 mm fully threaded screws to identify its chances and limitations. In this retrospective study, data from patients who underwent IPTSFC1/C2 were analyzed. 23 patients (17 females and 6 males) with an average age of 73.1 years (y) were included. Mean VAS decreased significantly from preoperative 3.9 ± 1.8 to the last follow-up 2.6 ± 2.5 (p = 0.020) and neurological functions were preserved. In the radiological follow-up, we saw a single malposition of an inserted screw (2.27%) and one single bony fusion (4.54%). However, in 6 of 7 patients (85.71%), there was a loosening of the inserted screws due course. We demonstrated that the use of 3.5 mm fully threaded screws for IPTSFC1/C2 results in low rates of osseous fusions between C1 and C2. Therefore, their use in IPTSFC1/C2 is not suitable, especially for geriatric patients with impaired bone status.

## Introduction

Fractures of the upper cervical spine are a major challenge for surgeons. The region around C1 and C2 and especially the course of the vertebral arteries are subject to high anatomical variance^1,2^. Magerl's transarticular screw fixation of C1 and C2 is a safe technique to achieve a stabilization of the atlantoaxial joint (AAJ) leading to solid bony fusion^3^. In Magerl's transarticular screw fixation, the skin incision is made in the neck and the laminae of C1–C7 are exposed. This is followed by bilateral transarticular screw fixation through the lateral mass of C1 and C2. To achieve high spinal fusion rates, autologous bone is placed in the joints and between the posterior arches of C1/C2^3–5^. Because autologous bone must be harvested mostly at the iliac crest and the laminae of C1–C7 must be exposed, Magerl's transarticular screw fixation is associated with increased operative trauma^3^. This can be a limiting factor in care, especially in aged patients with preexisting conditions. For such a patient population, minimally invasive surgery (MIS) can play a critical role in care and have a significant impact on the outcome. Many authors have addressed minimally invasive techniques for spinal fracture repair in the past^6–9^. The technique described by Magerl et al. has frequently been modified and techniques for performing the procedure in a minimally invasive manner have been developed^10–14^. In some of the minimally invasive procedures, in addition to percutaneous screw fixation, dorsal spondylodesis is established in a mini open procedure^10,12,14^. However, some authors have demonstrated isolated percutaneous techniques without an additional spinal fusion^11,14^. We also performed isolated percutaneous transarticular screw fixation of C1 and C2 (IPTSFC1/C2) without establishing a spinal fusion by bone grafting in selected cases. We hereby aim to present a retrospective study looking at 23 patients who underwent IPTSFC1/C2 to highlight the safety and efficacy of this procedure.

## Results

A total of 23 patients (17 female and 6 male) who were treated at our hospital from 2008 to 2012 using IPTSFC1/C2, were included in this study. The mean age was 73.1 ± 10.9 y at operation and 74.1 ± 10.9 y at the last follow-up. The median follow-up time was 6 months (range 0–52; Q1/Q3 1/12). The clinical data of the patients are stated in Table 1.

**Table 1:** Clinical data.

Patient	Age (in y)	Gender	Follow-up (in mo)	Frankel pre OP	Frankel post OP	Frankel LFU	VAS pre OP	VAS post OP	VAS LFU
1	73	m	6	E	E	E	3	0	7
2	66	m	6	E	E	E	1	5	0
3	57	f	1	E	E	E	6	7	6
4	84	f	3	E	E	E	2	8	2
5	57	f	10	E	E	E	3	0	0
6	67	m	1	C	D	D	6	3	0
7	84	f	12	E	E	E	3	2	0
8	55	f	10	E	E	E	X	X	X
9	90	f	12	E	E	E	3	8	0
10	74	f	1	E	E	E	5	2	2
11	92	f	1	E	E	E	X	X	X
12	78	m	0	E	E	E	7	2	2
13	81	f	32	E	E	E	X	2	5
14	76	f	0	E	E	E	X	X	X
15	79	f	30	E	E	E	X	X	2
16	70	f	6	E	E	E	X	5	X
17	63	f	36	E	E	E	6	2	0
18	84	f	12	E	E	E	3	3	2
19	60	f	52	E	E	E	3	X	X
20	75	f	42	E	E	E	X	X	X
21	79	m	3	E	E	E	X	X	X
22	58	m	12	E	E	E	X	X	X
23	79	f	4	E	E	E	X	6	6

*y* years, *m* male, *f* female, *mo* months, *OP* operation, *LFU* last follow-up, *VAS* visual analogue scale, *X* no value available.

**Figure 1:**
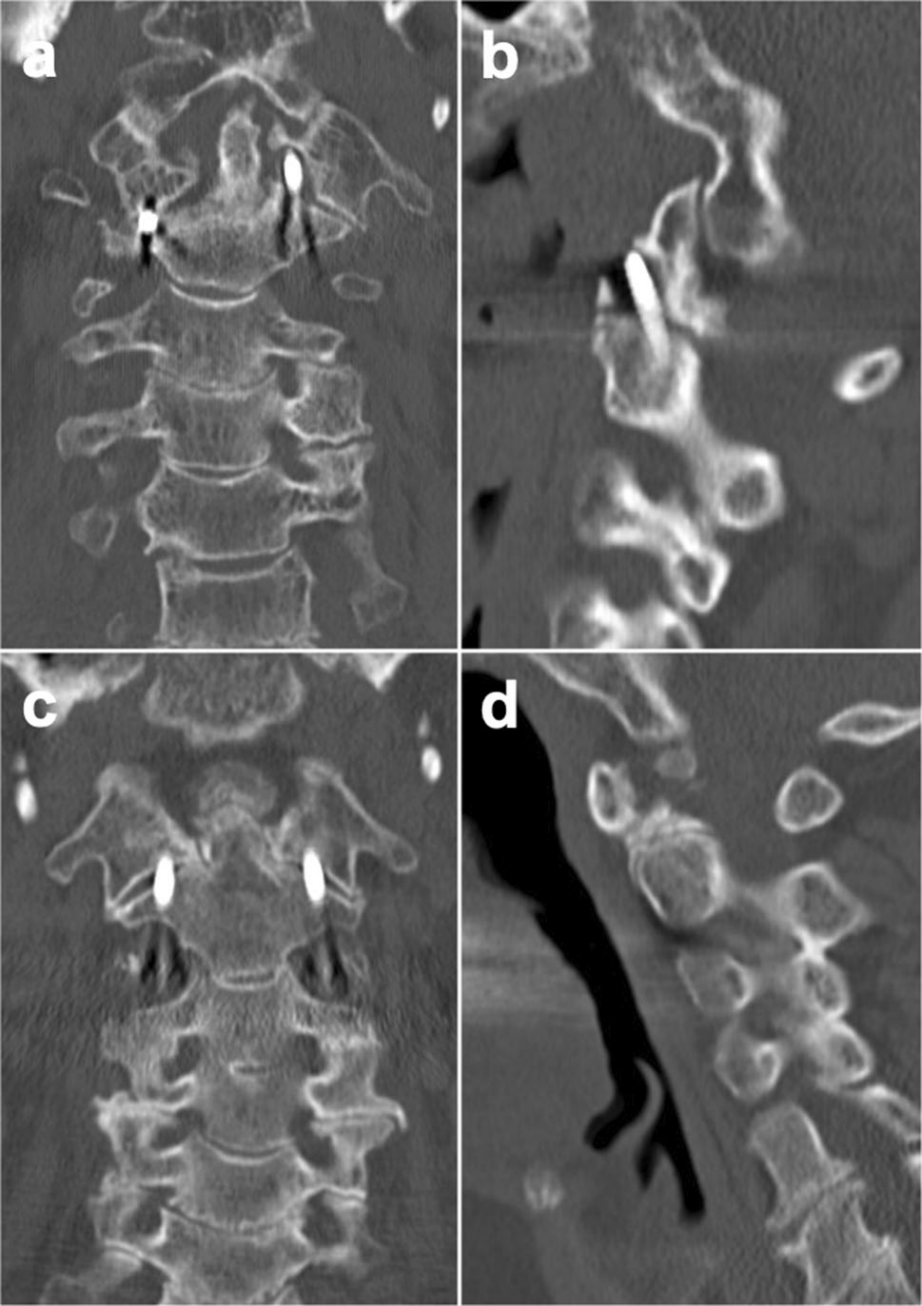
Material loosening versus spinal fusion. This figure shows the radiological outcome of two patients on computed tomography (CT) > 120 d postoperatively. (**a**) Patient 1 coronal plane: In the cranial region of the left-sided transarticular inserted screw, a clear lysis zone can be seen within the lateral mass of C1 as a correlate of material loosening. (**b**) Patient 1 sagittal plane: Also, in the sagittal plane, the loosening of the screw in the left-sided lateral mass of C1 can be clearly traced. (**c**) Patient 2 coronal plane: In this patient, no material loosening can be identified in the coronal plane. In the area of the right-sided joint space of C1/C2, a bony fusion can be identified. (**d**) Patient 2 sagittal plane: Here, too, the bony fusion between C1 and C2 can be traced.

**Figure 2:**
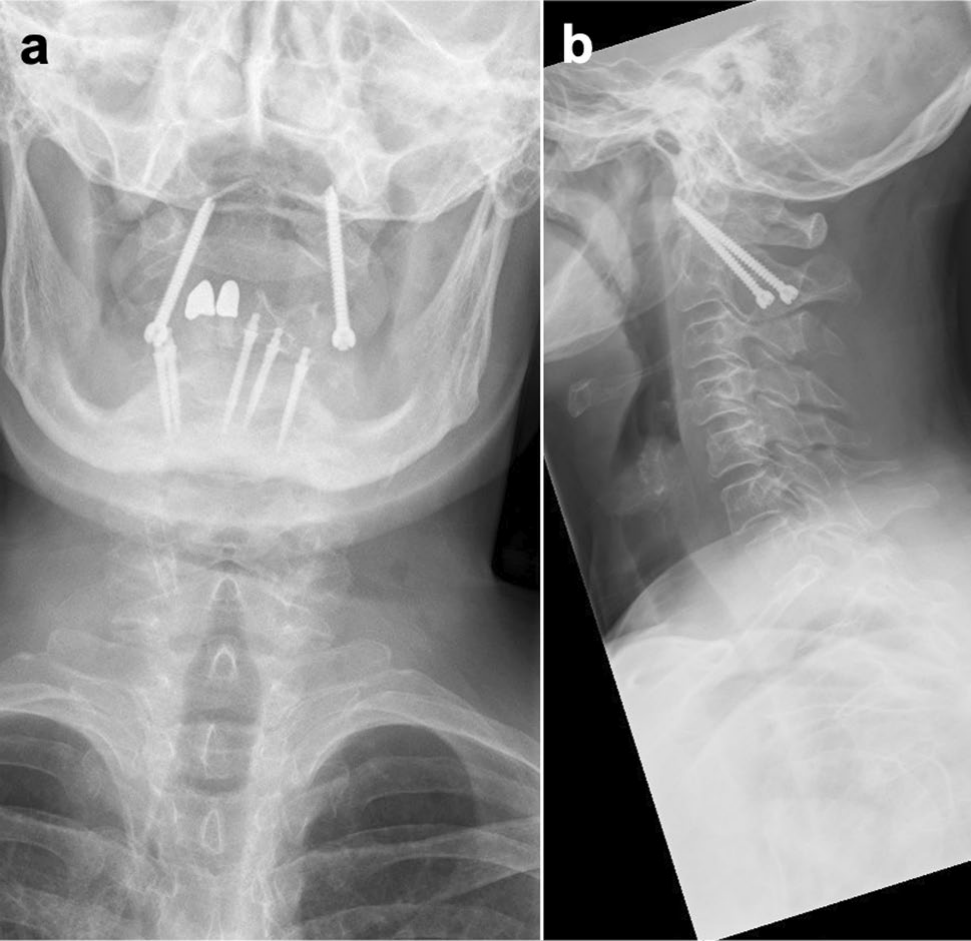
Incorrect screw position. This figure shows the malpositioning of a screw that occurred in the current study**.** Conventional X-ray. (**a**) Anterioposterior plane. The right-sided screw misses the articular surface of C1 on the medial side. No bridging of the joint occurs and the screw does not find a position in the right lateral mass of C1. (**b**) Sagittal plane. In this plane, the screw position appears largely correct.

Preoperatively, 22 patients did not show any neurological disorders and thus showed an E on the Frankel scale. One patient presented with a C on the Frankel scale, due to cerebral ischemia in the medical history. Postoperatively, 22 patients showed stable neurological functions with an E on the Frankel scale. One patient showed an improvement in neurological functions from Frankel C to Frankel D. The mean preoperative VAS was 3.9 ± 1.8, the mean postoperative VAS was 3.7 ± 2.6 and the mean VAS at last follow-up was 2.6 ± 2.5. The difference in the mean values of VAS preoperatively and at last follow up was statistically significant (p = 0.020), while the difference in the mean values of VAS preoperatively and postoperatively were not significant (p = 0.651). Perioperative complications were urinary tract infections (n = 5), falls (n = 1), Clostridium difficile diarrhea (n = 1) and exitus lethalis due to nosocomial pneumonia and sepsis (n = 1). The mean postoperative stay in the ICU was 1.4 ± 1.5 days (d). The mean postoperative in hospital stay was 10 d ± 5.68 d. Of the 23 patients treated with IPTSFC1/C2, 14 patients presented with an unstable fracture, five with pseudarthrosis after Böhler’s screw fixation, three with osteolysis due malignancy and one with AAI in rheumatoid arthritis. The indication for operation of each patient are stated in Table 2. Relevant secondary diagnoses were arterial hypertension (n = 12), malignancy (n = 4), prior cardiac disease (n = 4), alcohol abuse (n = 2), ischemic cerebral insult (n = 2), prior pulmonary disease (n = 2), epilepsy (n = 1), renal failure (n = 1), dementia (n = 1) and previously known osteoporosis (n = 1). Seven (31.82%) of the patients received a CT scan > 120 d after surgery. In these patients, spinal fusion between C1 and C2 was evident in only one case (14.29%) (Fig. 1c,d). In all these patients, correct implant placement was documented by the immediate postoperative CT scan; however, in six of these patients (85.71%), loosening of the implants was seen at follow up (Fig. 1a,b). In the entire follow-up patient population, there was a single malposition of one of the inserted screws (2.27%) (Fig. 2a,b). Surgical revision was not necessary in any case. The radiological data are stated in Table 2.

**Table 2:** Radiological data and indication for surgery.

Patient	Indication for surgery	Fusion	Correct screw placement	Screw loosening	Radiography > 120 d post OP
1	combined JF and DF II	n	y	n	n
2	DF II	n	y	y	y
3	instable osteolytic lesion	n	y	n	n
4	combined EF II and DF II	n	y	n	y
5	instable osteolytic lesion	n	y	y	y
6	IPA after DF II	n	y	n	n
7	IPA after DF II	n	y	y	y
8	IPA after DF II	n	y	n	y
9	BF III	n	y	n	n
10	instable osteolytic lesion	n	y	n	n
11	BF III	n	y	n	n
12	BF III	n	y	n	n
13	IPA after DF II and ASF	n	y	y	y
14	GF II and DF II	X	X	X	n
15	AAI in RA	y	y	n	y
16	GF II	n	n	n	n
17	EF I	n	y	y	y
18	BF III	n	y	y	y
19	IPA after DF II and ASF	n	y	y	n
20	BF III	n	y	y	y
21	GF II	n	y	y	y
22	BF III	n	y	n	y
23	EF I	n	y	n	y

*JF* Jefferson fracture, *DF* Dens axis fracture (Anderson and D’Alonzo), *BF* Benzel fracture, *EF* Effendi fracture, *IPA* instable pseudarthrosis, *ASF* anterior screw fixation, *GF* Gehweiler fracture, *AAI* atlantoaxial instability, *RA* rheumatoid arthritis, *d* days, *n* no, *y* yes, *X* no value available.

## Discussion

The retrospectively collected data of the above-described cohort revealed that IPTSFC1/C2 is a safe surgical technique, as already described^11,14^. Performed by experienced surgeons it is associated with only rare implant malpositioning, few perioperative complications, and low revision rates. However, due to the high anatomic variance, preoperative screening of patients for the presence of HRVA is necessary. If contraindications are ruled out, the IPTSFC1/C2 represents a treatment option for patients with high-grade AAI who are multimorbid, have a low life expectancy and cannot bear the risk of open surgical procedures. We demonstrated that after IPTSFC1/C2 no revision surgeries were required, no neurological deficits recurred, and only 1 out of 44 screws (2.27%) was malpositioned. To prevent dislocation in the AAJ and thus the occurrence of complications such as peripheral neurological functional deficits, autonomic dysfunction, immobility, and death, IPTSFC1/C2 appears to be an appropriate therapeutic option. For a patient population as mentioned above, IPTSFC1/C2 may represent a safe surgical treatment option with the smallest possible surgical trauma to enable rapid remobilization and dehospitalization. Our data agree with those found in the literature on this subject^12–14^. The advantage of percutaneous transarticular screw fixation over the classic transarticular screw fixation of C1 and C2 as an open approach is the significantly lower surgical invasiveness^10^. IPTSFC1/C2 provides a minimally invasive treatment option for patients who have a contraindication to treatment with classic anterior screw fixation according to Böhler or in whom it has failed. This is especially true in aged patients with reduced bone density, where Böhler’s screw fixation is known to have high rates of implant loosening and nonunion^15,16^. The IPTSFC1/C2 as another minimally invasive therapy option available, seems to be quite worthwhile. Furthermore, it has been shown that minimally invasive percutaneous transarticular screw fixation of C1/C2 combined with a mini open technique to establish spinal fusion resulted in fusion rates of 97.5 to 98%^12,13^. These rates were similar to spinal fusion rates achieved with classic transarticular screw fixation with dorsal bone grafting between C1 and C2^3,17^.

However, in the current study, we were able to show that IPTSFC1/C2 using 3.5 mm fully threaded screws, in geriatric patients, is not a suitable option if the goal of the treatment is spondylodesis. We saw spinal fusion between C1 and C2 in only 1 (14.29%) and implant loosening in 6 out of 7 (85.71%) patients that were followed up by CT scans > 120 d postoperatively. Our data show that in most patients, an osseous fusion between C1 and C2 cannot be achieved by IPTSFC1/C2 using fully threaded screws, which leads to loosening of the implants over time and potentially to a renewed AAI. Thus, successful achievement of a long-term stabilization of the AAJ depends on the achievement of spinal fusion between C1 and C2. Only temporal limited AAI as osteolytic lesions or fractures with the potential of regaining osseous stability may be suitable for the usage of fully threaded screws in terms of IPTSFC1/C2. In other patients, it seems essential to use partially threaded screws or surgically establish an apposition of autologous cancellous bone to achieve osseous fusion and achieve long-term stability.

In a retrospective study, Lvov et al. compared patients treated for traumatic injury to the upper cervical spine using percutaneous transarticular stand-alone screws or supplemental incision of the midline and spinal fusion. All patients in whom spinal fusion was surgically established showed a solid fusion of C1 and C2 over time. Surprisingly, Lvov et al. also postulated spinal fusion in 14 of 15 (93.33%) patients who were treated with a stand-alone screw^14^. These data contradict the data we generated with a fusion rate of 14.29%. With a similar surgical technique compared to our current study, without performing active measures to establish a bony fusion, the reasons for these different results are unlikely to be due to the surgical technique. However, an important difference in the study performed by Lvov et al. lies in the type of screws used. While Lvov et al. used 4 mm titanium lag screws, only 3.5 mm titanium fully threaded screws were used in the current study. Thus, the type of screws could be the reason for the different fusion rates. Another study on the long-term outcome after transarticular instrumentation with stand-alone screws, in which only lag screws were used, also showed high fusion rates of 93.1% without further active intraoperative spondylodesis^18^. Furthermore, risk factors for screw-associated complications after posterior C1/C2 fixation were named in a review article in the past^19^. It was shown that the highest rate of failure was observed with the use of fully threaded screws, whereas no failure was observed with the use of lag screws. Thus, it was concluded that the use of 3.5 mm or thinner fully threaded screws is a risk factor for screw-associated complications, such as material loosening and failure to fuse^19^.

In concordance with the literature, our data clearly show that usage of fully threaded 3.5 mm screws for IPTSFC1/C2 is not a suitable therapeutic option when long-term stability and spinal fusion between C1 and C2 is the goal of surgical therapy. Due to the high mobility of the upper cervical spine, fully threaded screws seem to not provide enough rigidity to achieve spinal fusion. An efficient interdisciplinary cooperation is required to establish indication for IPTSFC1/C2 and select the appropriate patients for this procedure, especially as a small additional incision in the midline to perform an additional fusion does extend the operation only slightly. If an isolated percutaneous approach cannot be avoided, 3.5 mm fully threaded screws should not be used in IPTSFC1/C2.

All in all, minimally invasive surgical techniques can be used to provide surgical therapy in patients who would otherwise require conservative therapy with potentially worse outcomes and higher risk of complications e.g. soft tissue lesion due to rigid cervical orthosis. Therefore, it seems worthwhile to highlight the limitations of currently available surgical techniques to establish the path for the development of new, more effective and less invasive surgical procedures or implants such as anterior plates for odontoid fractures^20–22^. In the future, further research should be conducted on minimally invasive techniques for the safe management of AAI, as AAI in multimorbid patients will continue to increase in frequency and clinical relevance in the context of demographic change and a rapidly aging population.

Some of the patients we cared for were prematurely lost to follow up and CT imaging was not available > 120 d postoperatively. Thus, in these patients the radiological outcome could only be followed up to a limited extent. The relatively small patient collective also represents a limitation of this study. However, it must be said that IPTSFC1/C2 was performed in our hospital only in very selected cases due to the very strict indication and therefore the patient population is correspondingly small.

## Conclusion

IPTSFC1/C2 represents a technically safe technique for the surgical management of AAI. However, in the current study, we could show that the use of 3.5 mm fully threaded screws for this procedure, results in low rates of osseous fusions between C1 and C2. Therefore, the use of these screws, as stand-alone screws, is not suitable for geriatric patients with impaired bone status and a dorsal apposition of autologous cancellous bone between C1 and C2 should be performed to establish spinal fusion. If an isolated percutaneous approach cannot be avoided, 3.5 mm fully threaded screws should not be used.

## Patients and methods

This study was reported according to the Strengthening The Reporting of Observational studies in Epidemiology (STROBE) guideline^23^. All methods were carried out in accordance with relevant guidelines and regulations. This study and its protocols were approved by the ethics committee of the Hamburg medical association. The patient data has been anonymized and therefore, according to the Hamburg medical association, informed consent was not needed (ethics vote WF-008/21).

We hypothesize that usage of fully threaded 3.5 mm titanium screws for IPSFC1/C2 is not suitable for establishing a spinal fusion between C1 and C2, in geriatric patients.

### Patient population

Reviewing all posterior cervical operations at our institution in the time span from 2008–2012 we could identify 23 patients matching the inclusion criteria of an IPTSFC1/C2. The indication for surgical treatment was strictly determined in preoperative expert rounds. Indications were AAI after conservative therapy failure, unstable fractures, progressive osteolytic processes resulting in instability, or failure of other surgical therapy, such as anterior screw fixation. The respective indication for surgery for each patient is shown in Table 2. We performed a retrospective analysis of patients’ records and all available radiologic examinations in this regard.

### Operative technique

Preoperatively, extensive diagnostics including cross-sectional imaging via computed tomography (CT) and magnetic resonance imaging (MRI) with visualization of the vertebral arteries is necessary to rule out contraindications such as the presence of a high riding vertebral artery (HRVA) or hyper kyphosis.

**Figure 3:**
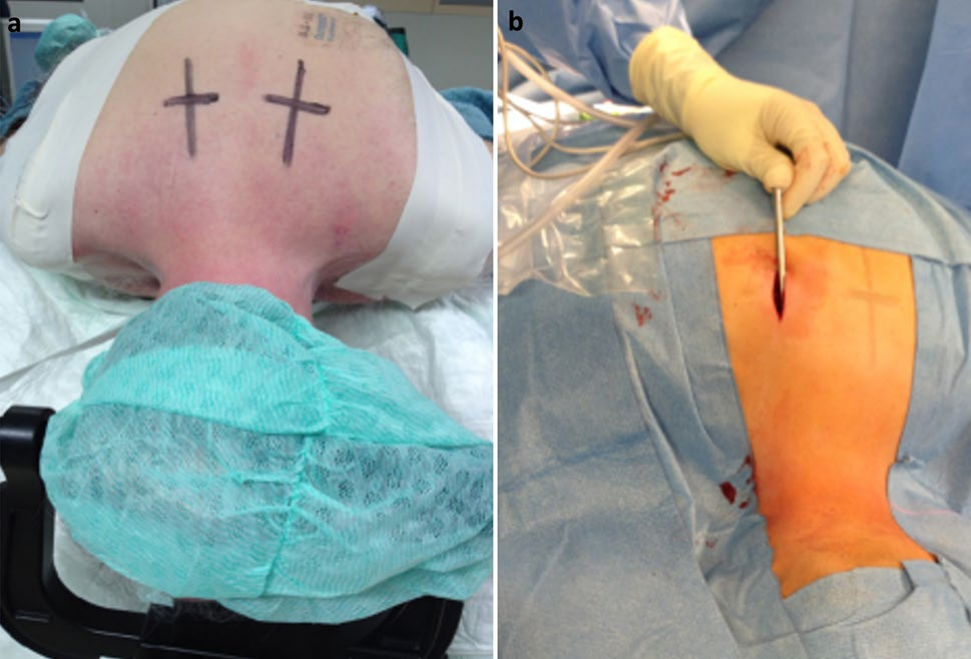
Intraoperative setting. After successful team time-out, a Mayfield clamp was applied, and the patient was positioned in a modified prone position, the so-called Concorde position. (**a**) First, the correct entry point for the skin incision is located with the aid of a K-wire and marked, usually at the level of TH 3/4 approximately 3 cm to the midline. (**b**) Subsequently, a 2 cm skin incision is made, and the fascia is opened. The drill guide is now advanced thrue the para spinal muscles and is placed on the lateral mass of C2. This is followed by fluoroscopically controlled drilling with constant monitoring for bone contact with the drill. When the drilling depth is sufficient according to the x-ray, the instrument provides the possibility of measuring the screw length. The screw can then be inserted through the guide instrument, again under fluoroscopic control.

The operation was performed under general anesthesia. Fiberoptic awake intubation was performed due to the present cervical instability. In all cases, the C1/C2 Access System from DePuy Synthes (Oberdorf, Switzerland) and 3.5 mm, fully threaded, titanium alloy, cortical screws were used. A Mayfield clamp was applied, and the patient was positioned in a modified prone “concorde” position^24^. Fluoroscopic control of the anatomically correct position of C1 and C2 and in fracture cases verification of successful reduction, was applied. Sterile washing and draping according to the protocol followed. First, the correct entry point for the skin incision was located with the aid of a K-wire and marked (Fig. 3a). The K-wire was then placed percutaneously along the previously determined trajectory at the entry point for the screw, also under fluoroscopic control, on the C2 lamina, with help of a Jamshidi needle. The entry point for the screw is approximately 2 mm craniolateral to the medial edge of the caudal process of C2. After correct placement of the K-wire, the cortical bone of the lamina was punched with the K-wire to prevent slippage. Subsequently, a 2 cm skin incision was made, and the fascia was opened. A guide sleeve and then the tissue protection sleeve for the drill were passed over the K-wire to the entry point of the screw onto the lamina of C2 (Fig. 3b). From this point on, it was essential that the tissue protection sleeve always remained in firm bone contact and that the positioning at the entry point of the screw and the trajectory were not altered. If a dislocation occurred, removal of the sleeve, revisiting the screw entry point with the K-wire, and repeating the previous steps were necessary. If the sleeve remained securely on the lamina at the entry point, the screw channel was opened meticulously using a twist drill under fluoroscopic control. It was essential to keep the drill straight in the axis to avoid bending or breaking the drill and cutting osteoporotic bone. Therefore, an accurate fluoroscopic setting of the subsequent screw trajectory had to be set and constrained before the drill entered the bone. Once the drill entered the bone, no further correction could take place to avoid the above complications. Changes could only be achieved by complete repositioning. Once the screw channel had been opened successfully, the screw length was determined using the marking on the drill bit. In the case of particularly firm or sclerotic bone, this was followed by cutting a thread in the screw channel via the tissue protection sleeve, which remained permanently with firm bone contact at the entry point, also under fluoroscopic control. The 3.5 mm fully threaded screw was then inserted into the prepared screw channel with the previously determined length, using a self-retaining screwdriver, through the tissue protection sleeve under fluoroscopic control. Subsequently, the same procedure was followed for positioning the contralateral screw. Final fluoroscopic control of the screw position. After rinsing of the incisions, the wound was closed in layers with the reconstruction of the fascia, adapting subcutaneous suture and suture of the skin. Patients received a soft cervical orthosis for 6 weeks postoperatively. Post op cervical spine CTs were used to check for successful reduction and implant positioning.

### Clinical and radiological evaluation

**Figure 4:**
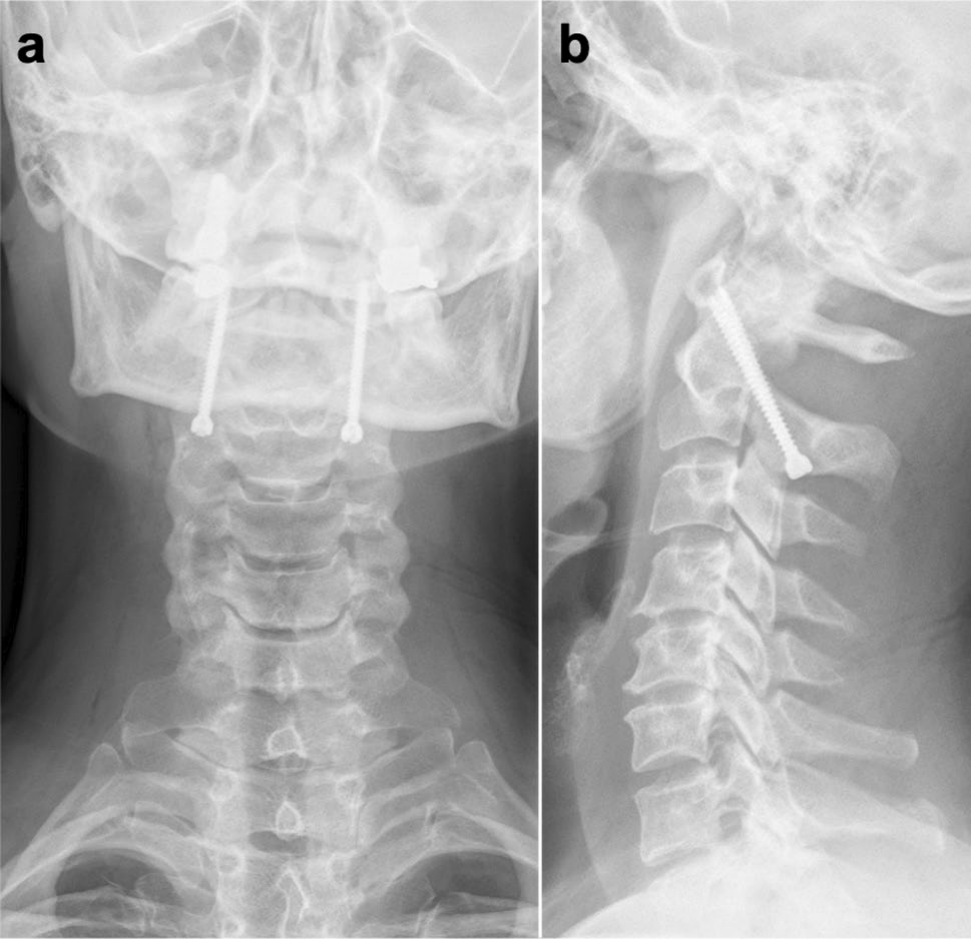
Correct screw position. This figure shows a typical example of a correct material position after isolated percutaneous transarticular screw fixation of C1 and C2 (IPTSFC1/C2). Conventional X-ray. (**a**) Anterioposterior plane. The screws inserted bridge the atlantoaxial joints on both sides and pass thrue the lateral mass of C1 and C2 on both sides. (**b**) Sagittal plane. The screws do not protrude more than 5 mm beyond the anterior arch of C1. If all these criteria match, the position of the screw is considered to be correct.

Preoperative and all postoperative imaging, consisting of conventional radiography (CR) and CT, were evaluated by the first and last author independently. Questionable cases were reevaluated by the spine surgeon M.S. Postoperative imaging was evaluated for implant positioning, possible implant loosening and the presence of spinal fusion. As reported by Kaminski et al.^4^ we evaluated the implant positioning according to the criteria published by Madawi et al.^25^. The implants position was considered appropriate if the screws were bridging the atlantoaxial joints on both sides, passing the lateral mass of C2 and C1 on both sides and did not protrude more than 5 mm beyond the anterior arch of C1 (Fig. 4a,b). Screws that did not meet all criteria were considered to be malpositioned. If there was a lightening halo around the screws on the follow up CR or CT scans, we assumed implant loosening. The presence of spinal fusion was assumed if there was radiographically clear bony bridging between C1 and C2. The following parameters were collected to evaluate the clinical outcome of the patients. Postoperative hospitalization time and time in the ICU were recorded. Furthermore, we evaluated the patients' pain symptoms pre- and postoperatively by means of a 10-point visual analogue scale (VAS). Peripheral neurological assessment of the patients was performed pre- and postoperatively as well as at last follow-up by means of the Frankel scale^26^.

### Statistical analyses

For statistical analysis, the software SigmaPlot 13 of Systat Software Inc., San Jose, CA, USA was used. The analysis of the patient data was descriptive. Continuous variables are expressed as mean ± standard deviation. Exceptions are found in the values of follow-up time. These values are given in median, range, and quartiles (Q_1_/Q_3_). Categorial variables are expressed as number and/or percentage. The Shapiro–Wilk test was used to test normal distribution. To evaluate the statistically significant differences between the preoperative and follow-up measurement time point, t-test for dependent samples in the case of a normal distribution or the Wilcoxon rank sum test in the case of a non-normal distribution was used. The significance level was *p* < 0.05.

### Ethics declaration

This study was reported according to the Strengthening The Reporting of Observational studies in Epidemiology (STROBE) guideline^23^. All methods were carried out in accordance with relevant guidelines and regulations. This study and its protocols were approved by the ethics committee of the Hamburg medical association. The patient data has been anonymized and therefore, according to the Hamburg medical association, informed consent was not needed (ethics vote WF-008/21).

## Data Availability

The data presented in this study are available within this article. All data generated or analyzed during this study are included in this published article. A summary of the data can also be provided upon request.

## References

[1] Goel A (2015). Craniovertebral junction instability: A review of facts about facets. Asian Spine J..

[2] Yin QS, Wang JH (2015). Current trends in management of atlantoaxial dislocation. Orthop. Surg..

[3] Magerl F (1987). Stable posterior fusion of the atlas and axis by transarticular screw fixation. Cerv. Spine.

[4] Kaminski A, Muhr G, Müller EJ (2008). Die transartikuläre C1-C2-Verschraubung. Unfallchirurg.

[5] Bahadur R, Goyal T, Dhatt SS, Tripathy SK (2010). Transarticular screw fixation for atlantoaxial instability—modified Magerl's technique in 38 patients. J. Orthop. Surg. Res..

[6] Koreckij T, Park DK, Fischgrund J (2014). Minimally invasive spine surgery in the treatment of thoracolumbar and lumbar spine trauma. Neurosurg. Focus.

[7] Dreimann M, Stangenberg M, Eicker SO, Frosch KH, Viezens L (2020). Minimally invasive posterior and anterior stabilization of the thoracolumbar spine after traumatic injuries. Unfallchirurg.

[8] Holly LT, Foley KT (2006). Percutaneous placement of posterior cervical screws using three-dimensional fluoroscopy. Spine.

[9] Hansen-Algenstaedt N (2015). Accuracy and safety of fluoroscopic guided percutaneous pedicle screws in thoracic and lumbosacral spine: A review of 2000 screws. Spine.

[10] Blauth M, Richter M, Lange U (1999). Transarticular screw fixation C1/C2 in traumatic atlantoaxial instabilities. Comparison between percutaneous and open procedures. Orthopade.

[11] Dimitriou J, Garvayo M, Coll JB (2020). Minimally invasive posterior percutaneous transarticular C1–C2 screws: how I do it. Acta Neurochir. (Wien).

[12] Dusad T (2018). Minimally invasive microscope-assisted stand-alone transarticular screw fixation without gallie supplementation in the management of mobile atlantoaxial instability. Asian Spine J..

[13] ElSaghir H, Boehm H, Greiner-Perth R (2005). Mini-open approach combined with percutaneous transarticular screw fixation for C1–C2 fusion. Neurosurg. Rev..

[14] Lvov I (2019). Minimally invasive posterior transarticular stand-alone screw instrumentation of C1–C2 using a transmuscular approach: Description of technique, results and comparison with posterior midline exposure. World Neurosurg..

[15] Andersson S, Rodrigues M, Olerud C (2000). Odontoid fractures: High complication rate associated with anterior screw fixation in the elderly. Eur. Spine J..

[16] Collins I, Min WK (2008). Anterior screw fixation of type II odontoid fractures in the elderly. J. Trauma.

[17] Grob D, Jeanneret B, Aebi M, Markwalder TM (1991). Atlanto-axial fusion with transarticular screw fixation. J. Bone Joint Surg. Br..

[18] Lvov I (2019). A comparison of the long-term results of posterior transarticular stand-alone screw instrumentation and magerl technique in patients with traumatic atlantoaxial instability: Mean 5-year follow-up study with radiological and patient-rated outcomes assessments. World Neurosurg..

[19] Lvov I (2019). Potential intraoperative factors of screw-related complications following posterior transarticular C1–C2 fixation: A systematic review and meta-analysis. Eur. Spine J..

[20] Daniels AH (2012). Preliminary biomechanical proof of concept for a hybrid locking plate/variable pitch screw construct for anterior fixation of type II odontoid fractures. Spine.

[21] Hu Y (2016). A novel anterior odontoid screw plate for C1–C3 internal fixation: An in vitro biomechanical study. Spine.

[22] Platzer P, Eipeldauer S, Vecsei V (2010). Odontoid plate fixation without C1–C2 arthrodesis: Biomechanical testing of a novel surgical technique and comparison to the conventional screw fixation procedure. Clin. Biomech. (Bristol, Avon).

[23] von Elm E (2008). The strengthening the reporting of observational studies in epidemiology (STROBE) statement: Guidelines for reporting of observational studies. Internist (Berl).

[24] Viezens L, Sehmisch S, Weiser L, Dreimann M, Lehmann W (2019). Dorsal stabilization of C1/C2 modified according to Goel-Harms with C1 pedicle screws. Oper. Orthop. Traumatol..

[25] Madawi AA (1997). Radiological and anatomical evaluation of the atlantoaxial transarticular screw fixation technique. J. Neurosurg..

[26] Frankel HL (1969). The value of postural reduction in the initial management of closed injuries of the spine with paraplegia and tetraplegia I. Paraplegia.

